# Stadiengerechte Therapie des Keratokonus

**DOI:** 10.1007/s00347-021-01410-8

**Published:** 2021-06-28

**Authors:** B. Seitz, L. Daas, L. Hamon, K. Xanthopoulou, S. Goebels, C. Spira-Eppig, S. Razafimino, N. Szentmáry, A. Langenbucher, E. Flockerzi

**Affiliations:** 1 Klinik für Augenheilkunde und Hochschulambulanz, Universitätsklinikum des Saarlandes UKS, 66421 Homburg/Saar, Deutschland; 2 Dr. Rolf M. Schwiete Zentrum für Limbusstammzellforschung und kongenitale Aniridie, Universität des Saarlandes, Homburg/Saar, Deutschland; 3 Institut für Experimentelle Ophthalmologie, Universität des Saarlandes, Homburg/Saar, Deutschland

**Keywords:** ABCD-Grading-System, Kontaktlinsen, Riboflavin-UVA-Crosslinking, Perforierende Keratoplastik, Intrastromale Ringsegmente, Tiefe lamelläre Keratoplastik, ABCD grading system, Contact lenses, Riboflavin UVA cross-linking, Penetrating keratoplasty, Intracorneal ring segments, Deep lamellar keratoplasty

## Abstract

Der Keratokonus (KK) ist eine progrediente kegelförmige Hornhautvorwölbung, die eine parazentrale Verdünnung an der Kegelspitze verursacht und typischerweise beidseitig asymmetrisch auftritt. Nach einer sorgfältigen Anamnese und Einstufung des Schweregrades steht heute eine gezielte stadiengerechte Therapie zur Verfügung. Ist der Brillenvisus nicht mehr ausreichend, werden von einem Spezialisten formstabile sauerstoffdurchlässige Kontaktlinsen (KL) angepasst. Bei Progression und für den Patienten im Alltag nutzbarem Visus empfiehlt sich das Riboflavin-UVA-Crosslinking (CXL), bei herabgesetztem Visus und klarer zentraler Hornhaut sind bei KL-Intoleranz intrastromale Ringsegmente (ICRS) indiziert. Ist das Stadium weiter fortgeschritten, empfiehlt sich die tiefe anteriore lamelläre (DALK) oder perforierende Keratoplastik (PKP). Bei einem akuten Keratokonus ist die PKP kontraindiziert, allerdings verkürzen tiefstromale Nähte zur Readaptation des Descemet-Risses mit Gasfüllung der Vorderkammer den Verlauf erheblich. Fast keine andere Augenerkrankung ist heutzutage einer frühen apparativen Diagnose und stadiengerechten Therapie so gut zugänglich wie der KK.

## Abstract (en)

Keratoconus (KC) is a progressive cone-shaped corneal protrusion that causes paracentral thinning at the apex of the cone and typically occurs asymmetrically on both sides. After a careful anamnesis and classification of the degree of severity a targeted treatment appropriate to the stage of the disease is available. If the visual acuity is no longer sufficient, rigid gas-permeable contact lenses (CL) are fitted by a specialist. Riboflavin UVA cross-linking (CXL) is recommended in cases of progression and visual acuity that is still useful for the patient. Intracorneal ring segments (ICRS) are indicated for CL intolerance in cases of reduced visual acuity and a clear central cornea. If the stage is more advanced, deep anterior lamellar keratoplasty (DALK) or penetrating keratoplasty (PKP) is recommended. A PKP is contraindicated in acute KC but deep stromal sutures for readaptation of the Descemet tear with gas filling of the anterior chamber can considerably shorten the course. Almost no other eye disease is nowadays as easily accessible for an early instrument-based diagnosis and stage-appropriate treatment as KC.

## Lernziele

Nach Lektüre dieses Beitrags …

- ist Ihnen die Klassifikation des Schweregrades des Keratokonus (KK) nach dem ABCD-Grading-System nach Belin bekannt,
- sind Sie in der Lage, Ihre Patienten fundiert zur stadiengerechten Therapie zu beraten,
- ist es Ihnen möglich, die Indikationen für Kontaktlinsen, Crosslinking und intrastromale Ringsegmente kompetent zu trennen,
- kennen Sie die Indikation zur lamellären oder perforierenden Keratoplastik bei KK,
- können Sie Ihren Patienten eine zeitgemäße chirurgische Therapie des akuten Keratokonus empfehlen.

## Einleitung

**Figure 1:**
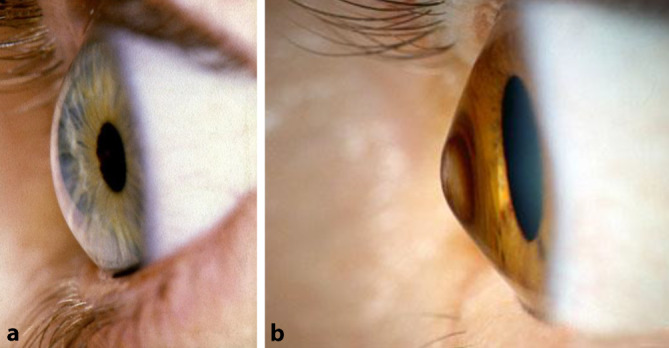

Der Keratokonus (KK) beginnt typischerweise in der Pubertät und geht mit einer oft progressiven, irregulären Vorwölbung und Verdünnung der Kornea an der Kegelspitze einher (Abb. 1). Die Folge ist eine bilaterale, häufig sehr asymmetrisch ausgeprägte zunehmende Ansteilung und Distorsion der Hornhaut mit Myopisierung und irregulärem Astigmatismus, bis hin zu oberflächlichen oder tiefen stromalen Vernarbungen und deutlich herabgesetztem Visus [1, 2, 3]. Ursächlich hierfür scheint eine Störung im Bereich der Verbindung der Kollagenfasern des kornealen Stützgerüstes zu sein, welches zu einer herabgesetzten biomechanischen Stabilität der Hornhaut führt. Der Prozess schreitet typischerweise bis zum 3. oder 4. Lebensjahrzehnt fort, wonach oft ein Stillstand eintritt. Die **Häufigkeit**

Häufigkeit

des Auftretens liegt bei 1:2000 in der europäischen Bevölkerung. Neuere Studien von Godefrooij et al. und Torres Netto verweisen auf eine höhere Häufigkeit von 1:375 in den Niederlanden [3] bzw. 1:21 bei Kindern und Jugendlichen in Saudi-Arabien [4].

Der KK hat eine komplexe **multifaktorielle Ätiologie**

multifaktorielle Ätiologie,

die noch nicht im Detail verstanden ist. Einen wichtigen Einfluss haben wahrscheinlich neben einem gestörten Kollagenstoffwechsel hormonelle, immunologische sowie Umwelt- und Verhaltensfaktoren (wie z. B. chronisches Augenreiben bei Allergie oder Neurodermitis) und multiple genetische Komponenten, die zu der Pathophysiologie dieser Erkrankung beitragen [5, 6, 7, 8, 9, 10]. Obwohl verschiedene Gen-Loci identifiziert worden sind (LOX, RAB3GAP1, ZNF469, TGFBI und COL5A1) [1], kam das International Committee for the Classification of Corneal Dystrophies (IC3D) zu dem Schluss, dass der KK zum jetzigen Zeitpunkt (noch) nicht als „Dystrophie“ eingeordnet werden kann [11]. Aktuelle Forschungsergebnisse weisen darauf hin, dass bei der Pathogenese des KK – ergänzend zum klassischen Lehrbuchwissen – auch entzündliche/immunologische Einflüsse eine Rolle spielen [12, 13].

Man unterteilt die verschiedenen **Ektasien der Kornea**

**Figure 2:**
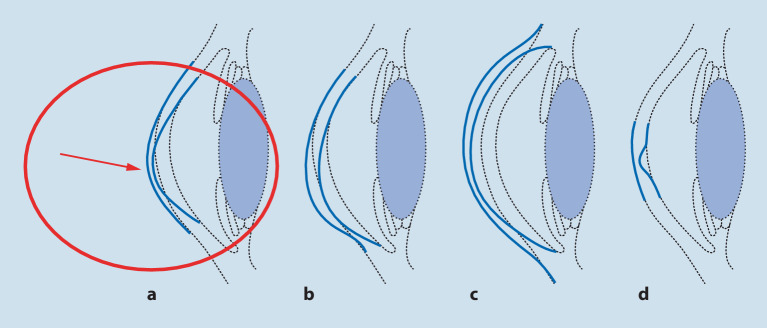

Ektasien der Kornea

in Keratokonus, Keratotorus (= pelluzide marginale Degeneration [PMD]), Keratoglobus und den sehr seltenen Keratoconus posticus circumscriptus (Abb. 2).

Die **Frühdiagnose**

Frühdiagnose

erfolgt heute bevorzugt in einer Zusammenschau topographischer, tomographischer und biomechanischer Kriterien [14, 15, 16, 17]. Dies ist besonders wichtig auch bei Kindern und Jugendlichen im Hinblick auf die familiäre Häufung des KK. **Fortgeschrittene Stadien**

**Figure 3:**
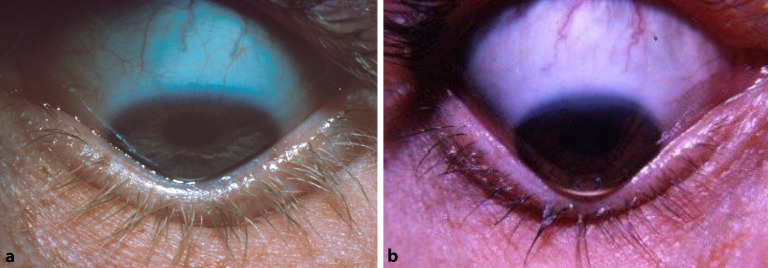

Fortgeschrittene Stadien

diagnostiziert man klinisch an der Spaltlampe durch parazentrale Stromaverdünnung, Vogt-Linien (wegdrückbare[!] meist senkrechte parallele Descemet-Fältelungen), Fleischer-Ring (oft inkomplette ringförmige epitheliale Eiseneinlagerungen durch Tränenfilmpooling an der Kegelbasis), prominente Hornhautnerven, subepitheliale Knötchen und/oder oberflächliche Narben, prädescemetale Narben (nach akutem Keratokonus = kornealer Hydrops) oder das Munson-Zeichen (benannt nach dem amerikanischen Augenarzt Edwin Sterling Munson [08.05.1870–02.02.1958]) (Abb. 3). Die konfokale Mikroskopie zeigt reproduzierbare strukturelle Veränderungen des subepithelialen Nervenplexus [18].

Durch die stetig verbesserte (besonders auch biomechanische) „**Frühestdiagnostik**

Frühestdiagnostik

“ ist die korrekte Diagnose des „einseitigen Keratokonus“ heute zur absoluten Rarität geworden. Meist handelt es sich bei klinisch unauffälligen Partneraugen um einen Forme-fruste-KK, der mit konventionellen tomographischen Diagnostikmethoden (noch) nicht zu erkennen ist [19, 20].

### Merke

Der Fleischer-Ring ist meist zentraler gelegen, als ihn der unerfahrene Untersucher erwartet, und er wird bevorzugt bei indirekter Beleuchtung sichtbar.

### Fallbeispiel

**Figure 4:**
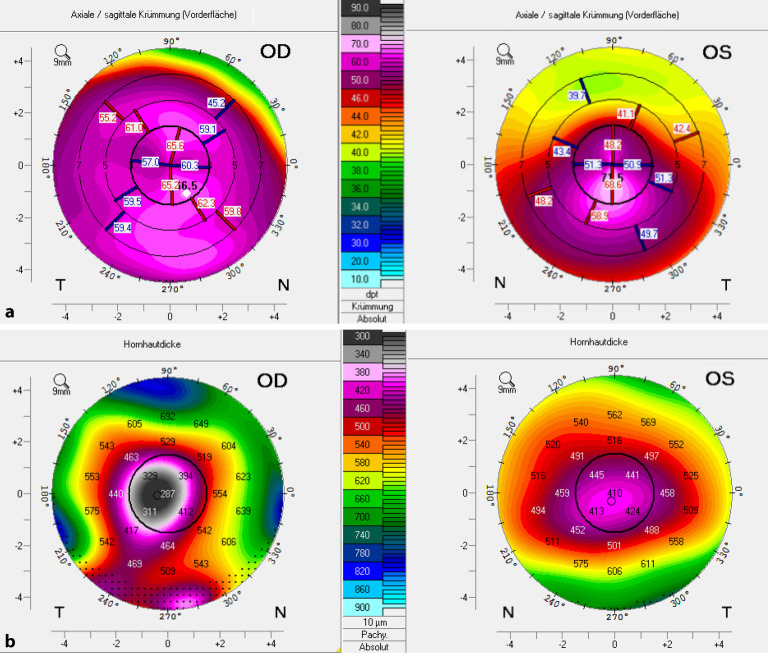

Ein 25-jähriger Patient stellte sich bei uns im Homburger Keratokonus Center (HKC) [21], in das wir seit dem Jahr 2010 mehr als 2000 Patienten eingeschlossen haben, mit beidseitiger Kontaktlinsen(KL)-Intoleranz vor. Der cum-correctione(cc‑)Brillenvisus betrug am rechten Auge (RA) 0,1 (Refraktion: −21,50/−2,75/A176°)/am linken Auge (LA) 0,2 (Refraktion: −6,00/−5,50/10°). Die mittlere Vorderflächenbrechkraft betrug am RA 61,3 dpt/LA 54,1 dpt (Abb. 4a), die mittlere Rückflächenkrümmung betrug am RA −11,2 dpt/LA −8,0 dpt. Die zentrale Dicke betrug am RA 287 µm mit starker prädescemetaler Vernarbung (bei Zustand nach akutem KK in der Anamnese vor 2 Jahren), am LA 410 µm ohne Narben (Abb. 4b). Vogt-Linien waren am RA vernarbungsbedingt nicht zu erkennen, am LA konnten sie ausgeschlossen werden. Am RA fand sich ein ausgeprägter Fleischer-Ring, am LA war er bei indirekter Spaltlampen(SL)-Beleuchtung nur inkomplett angedeutet. Wir empfahlen dem Patienten am RA eine zeitnahe Excimerlaser-assistierte perforierende Keratoplastik (PKP – 8,0/8,1 mm) mit doppelt fortlaufender Kreuzstichnaht nach Hoffmann in Vollnarkose unter stationären Bedingungen. Am LA empfahlen wir dem Patienten bei KL-Intoleranz und deutlich reduziertem Brillenvisus die Femtosekundenlaser-assistierte Implantation intrastromaler Ringsegmente in Tropfanästhesie unter ambulanten Bedingungen, zumal die mittelperiphere Hornhautdicke > 450 µm und das Zentrum narbenfrei waren. Hierdurch lässt sich typischerweise eine Steigerung des Sine-correctione(sc)- und cc-Visus und oft auch der KL-Toleranz erreichen. Gegen ein Riboflavin-UVA-Crosslinking (CXL) sprachen die reduzierte zentrale Hornhautdicke und die Kontaktlinsenintoleranz bei Brillenvisus von 0,2.

## Stadieneinteilung des Keratokonus

Insbesondere für die Vergleichbarkeit von Studienergebnissen ist die **Klassifikation**

Klassifikation

des Keratokonus in Stadien unerlässlich. Leider waren die vorgeschlagenen artifiziellen Klassifikationskriterien für die Stadieneinteilung des Keratokonus bisher zu keinem Zeitpunkt suffizient. Die **Amsler-Krumeich-Klassifikation**

Amsler-Krumeich-Klassifikation

[22, 23] bezieht keine topographischen oder gar tomographischen Kriterien mit ein. Insbesondere verwirrend bei diesen Klassifikationsversuchen war jedoch, dass nicht alle Detailkriterien wie „Myopie“, „Astigmatismus“, „zentrale Brechkraft“, „apikale Hornhautdicke“ oder das „Vorhandensein von Narben“ bei demselben Auge in demselben Keratokonusstadium resultierten.

Es gibt heutzutage eine Vielzahl an **Keratokonusindizes**

Keratokonusindizes

 – insbesondere für die Früherkennung und Klassifikation. Erfahrene Kliniker stützen ihre (Früh‑)Diagnose vornehmlich auf tomo- und topographische Indizes der Vorderabschnittstomographie oder -OCT (optische Kohärenztomographie). Seit Kurzem gewinnen auch die biomechanischen Parameter der Corneal Visualization Scheimpflug Technology zunehmend an Bedeutung. Aus dem Corvis Biomechanical Index (CBI) und dem Belin Ambrósio Display (BAD) wird der Tomographic Biomechanical Index (TBI) kreiert [17], der besonderes Potenzial für die „Frühestdiagnose“ des KK in der Sprechstunde für refraktive Chirurgie zeigt. Zuvor stand dem Kliniker der Keratoconus Match Index (KMI) als biomechanisches Kriterium zur Verfügung [15].

Die kürzlich von Belin und Duncan publizierte** ABCD-Klassifikation**

ABCD-Klassifikation

(Tab. 1) ist eine sinnvolle und übersichtliche Möglichkeit der Verlaufsdokumentation des KK [24, 25]. Diese beinhaltet eine Graduierung des „*A*nterior radius of curvature“ (A), des „*P*osterior radius of curvature“ = „*B*ack surface“ (B jeweils gemessen in einer 3,0-mm-Zone um die dünnste Stelle der Hornhaut), der „*C*orneal pachymetry at thinnest point“ (C) und der „*D*istance best-corrected vision“ mit Brille (D). Im Gegensatz zur klassischen Amsler-Krumeich-Klassifikation gibt es bei der ABCD-Klassifikation einen unterschiedlichen Schweregrad von 0 bis 4 für alle 4 Parameter. Ergänzt wird dabei ein „−“, wenn keine Narben zu sehen sind, „+“ für Narben, die die Irisdetails sichtbar lassen, und „++“ für Narben, die Irisdetails verdecken. Ein Beispiel dieser Klassifikation des KK wäre also: „A1, B2, C3, D2, +“. Wird diese Klassifizierung konsequent bei jeder Wiedervorstellung des Patienten, basierend auf den aktuellen Werten, angewandt und dokumentiert, so entsteht ein zeitlicher Verlauf dieser Buchstaben-Zahlen-Kombination, die mitunter schon auf den ersten Blick Aufschluss über eine stabile oder progrediente Verlaufsform geben kann.

**Table 1:** 

ABCD-Kriterien	A	B	C	D		Stadiengerechte Therapie
	Vorderflächenkrümmung (3-mm-Zone)	Rückflächenkrümmung (3-mm-Zone)	Dünnste Pachymetrie	Bestkorrigierter Brillenvisus	Vernarbung	
Stadium 0	> 7,25 mm (< 46,5 dpt)	> 5,90 mm (< 57,25 dpt)	> 490 µm	≥ 20/20 (≥ 1,0)	−	*KL, (CXL)*
Stadium I	> 7,05 mm (< 48,0 dpt)	> 5,70 mm (< 59,25 dpt)	> 450 µm	< 20/20 (< 1,0)	−, +, ++	*KL, CXL, (ICRS)*
Stadium II	> 6,35 mm (< 53,0 dpt)	> 5,15 mm (< 65,5 dpt)	> 400 µm	< 20/40 (< 0,5)	−, +, ++	*KL, CXL, ICRS*
Stadium III	> 6,15 mm (< 55,0 dpt)	> 4,95 mm (< 68,5 dpt)	> 300 µm	< 20/100 (< 0,2)	−, +, ++	*KL, (CXL), ICRS, DALK*
Stadium IV	≤ 6,15 mm (≥ 55,0 dpt)	≤ 4,95 mm (≥ 68,5 dpt)	≤ 300 µm	< 20/400 (< 0,05)	−, +, ++	*DALK, PKP, KS-Plastik*

*KL* Kontaktlinsen, *CXL* Crosslinking, *ICRS* intrakorneale Ringsegmente, *DALK* „deep anterior lamellar keratoplasty“, *PKP* perforierende Keratoplastik, *KS-Plastik* Korneoskleralplastik

## Therapieoptionen beim Keratokonus

Zu den Therapieoptionen zählen zum einen die Brille, weiche oder formstabile sauerstoffdurchlässige Kontaktlinsen. Zum anderen gibt es eine Reihe invasiver Optionen, wie z. B. die phototherapeutische Keratektomie (PTK), das Riboflavin-UVA-Crosslinking (CXL), die intrastromalen Ringsegmente (ICRS), tiefstromale sog. „Muraine-Nähte“ bei akutem Keratokonus und die Hornhauttransplantation – unterteilt in tief lamellär (DALK), perforierend (PKP) oder die zentrale Korneoskleralplastik [26, 27].

### Merke

Die Tab. 1 zeigt die vorgeschlagene Zuordnung der KK-Stadien in der ABCD-Klassifikation zu den Therapieoptionen.

Im Einzelfall mit zentral weitgehend regulärem Astigmatismus werden auch phake torische Kunstlinsen bei klarer Linse [28] und torische Kunstlinsen im Rahmen der Kataraktoperation bei stabilem KK diskutiert. Derartige Kunstlinsen können nur den regulären Anteil des Astigmatismus ausgleichen. Daher ist hier präoperativ eine hornhauttopographische Untersuchung zur Bestimmung des regulären Anteils des Astigmatismus – beispielweise mithilfe einer Fourier-Analyse – essenziell!

Zu den **Kontraindikationen**

Kontraindikationen

für einen chirurgischen Eingriff beim KK zählen alle radialen und zirkulären Keratotomien sowie „andere Keratotomien“ (wie z. B. nach Lombardi 1997 [29]) und die Laser in-situ Keratomileusis (LASIK). Als refraktivchirurgisches, laserablatives Verfahren wird die PRK vor/während/nach CXL vorgeschlagen [30]. Diese Methode hat allerdings wegen ihrer „Unberechenbarkeit“ keinen nennenswerten Einzug in das Stufentherapieschema des KK in Deutschland gefunden. Dasselbe trifft auch für die sog. „Bowman-Layer-Transplantation“ zu, die konzeptionell und technisch viele Fragen offen lässt [31].

### Brille

Der KK führt typischerweise sowohl zur Brechungs- als auch Achsenmyopie und zum zunehmend **irregulären Astigmatismus**

irregulären Astigmatismus.

Grundsätzlich können und sollten die Kurzsichtigkeit und der reguläre Anteil des Astigmatismus initial mit einer Brille ausgeglichen werden. Generell ist eine Brille aber auch als Alternative bei jedem KL-Träger wichtig. Zumindest können im Rahmen der optischen Versorgung von KK-Patienten mit einer „Besser-als-nichts-Brille“ bei Bedarf Karenzzeiten überbrückt werden. Ob ein **Brillenversuch**

Brillenversuch

ein Erfolg wird, hängt v. a. von der Lage des Apex, vom Grad der Hornhautirregularität, von der Qualität der Refraktion und der Erwartungshaltung des Patienten ab. Eine mehrtägige KL-Tragepause ist v. a. auch bei KK-Patienten vor einer neuen Brillenanpassung notwendig [32]. In jedem Fall sollten die Korrektionswerte in der Messbrille für mindestens 15 min probegetragen werden. Bei der Fassungswahl haben sehr kleine Brillenglasformen 2 Vorteile: Sie sind auch bei hohen Dioptrienwerten relativ leicht, gleichzeitig treten kaum prismatische Nebenwirkungen auf.

#### Merke

Mit einer „Besser-als-nichts-Brille“ können bei Bedarf Kontaktlinsenkarenzzeiten überbrückt werden.

### Kontaktlinsen

Optisch betrachtet, verursachen die ersten formstabilen KL für Patienten mit mäßigem oder fortgeschrittenem Keratokonus zumeist ein „Aha-Erlebnis“. Gleichzeitig ist es aber auch – zumindest beim ersten KL-Versuch – oft ein „tränenreiches“ Ereignis. Hat der Patient später mit der ermittelten Überrefraktion eine Vorstellung vom erreichbaren Visus, ist dies meist genug Motivation, um die Scheu und das anfängliche Fremdkörpergefühl zu überwinden.

#### Cave

Die Kontaktlinsenanpassung bei Keratokonuspatienten ist anspruchsvoll und setzt Erfahrung voraus.

Seit einigen Jahren werden auch weiche Silikon-Hydrogel-Keratokonuslinsen angeboten. Hierbei muss allerdings mit Visuseinbußen gegenüber der formstabilen KL gerechnet werden. Nach wie vor ist die O_2_-durchlässige, formstabile Kontaktlinsenversorgung die Methode der ersten Wahl [33]. KL-Hersteller, die sich der optischen Korrektur des Keratokonus verschrieben haben, bieten **formstabile Keratokonuslinsen**

formstabile Keratokonuslinsen

von einfachen rotationssymmetrischen über diverse torische Varianten bis hin zu asymmetrischen quadrantenspezifischen und multifokalen Geometrien an [32].

Bei schlechter Toleranz formstabiler KL kann das „**Huckepackprinzip**

Huckepackprinzip

“ angewandt werden, wobei unter der formstabilen Korrektionslinse eine weiche Trägerlinse als Polster dient [34]. Bezüglich der besseren Gasdurchlässigkeit ist man zwar geneigt, dafür **Silikonhydrogellinsen**

Silikonhydrogellinsen

zu verwenden, allerdings können diese unter Umständen mehr Epithelirritationen hervorrufen als klassische **Hydrogelmaterialien**

Hydrogelmaterialien

[32].

Eine weitere Alternative bietet die Versorgungen mit **Sklerallinsen**

Sklerallinsen

und insbesondere Minisklerallinsen, die sich auf der Sklera abstützen und eine fragile Hornhaut entlasten. Diese früher oft als überholt bezeichneten Linsen erfahren in den letzten Jahren wieder eine verstärkte Nachfrage und werden mittlerweile in bedarfsorientierten Formen, Größen und Geometrien aus gasdurchlässigem Material hergestellt [35]. Anpassaufwand, Effektivität und Risikoprofil erscheinen angemessen, insbesondere beim Keratoglobus und der fortgeschrittenen pelluziden marginalen Degeneration (PMD). Zuletzt sei noch die sog. „**Januslinse**

Januslinse

“ erwähnt, die einen Hybrid aus formstabilem Kern und weicher Hülle darstellt. Aufgrund der ungünstigeren Gasdurchlässigkeit sowie der hohen Produktionskosten hat sich dieser Typ allerdings in Deutschland nie etabliert [32].

#### Merke

Nach wie vor sind O_2_-durchlässige, formstabile Kontaktlinsen die erste Wahl.

Generell dienen KL nicht der Therapie des KK, sondern in erster Linie der **Visusrehabilitation**

Visusrehabilitation.

Manche Augenärzte vertreten die Auffassung, dass das konsequente Tragen von formstabilen Kontaktlinsen einer Progression vorbeugen würde. Heute mehren sich jedoch die Hinweise, dass das langjährige Tragen formstabiler Kontaktlinsen u. a. durch die Induktion proinflammatorischer Zytokine (wie z. B. IL[Interleukin]-6) die Progression begünstigen könnte. Moderne Sklerallinsen sollen diese potenziellen negativen Effekte nicht aufweisen [35].

In zahlreichen Publikationen sind perfekte **Fluoreszeinbilder**

**Figure 5:**
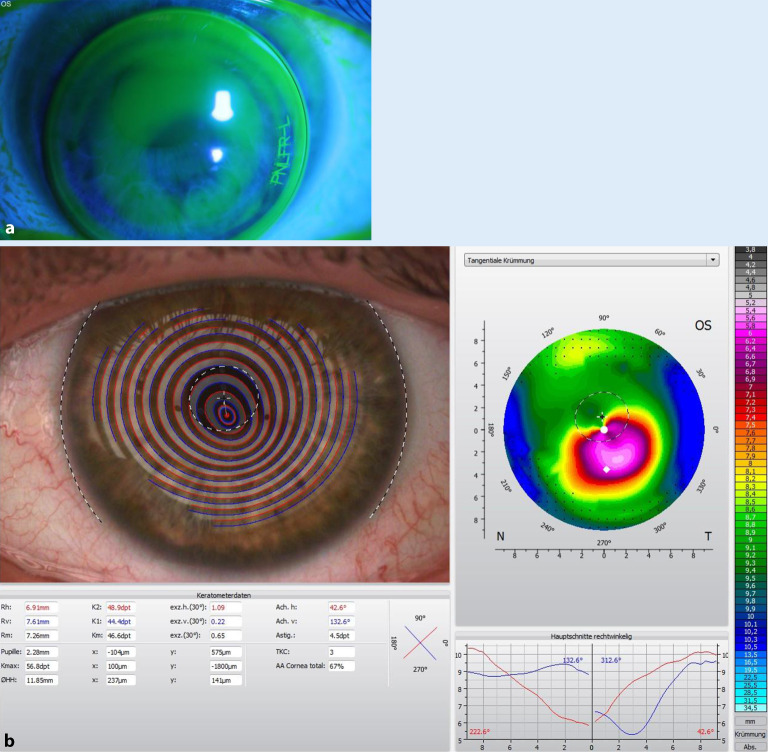

Fluoreszeinbilder

von ideal sitzenden Linsen zu sehen. Perfektion ist anzustreben, aber beim fortgeschrittenen KK nicht immer erreichbar. Solange der Patient die KL subjektiv gut verträgt, der individuelle Visusbedarf gedeckt ist und keine Hornhautirritationen bestehen, sind auch vom Ideal abweichende Fluoreszeinbilder „im grünen Bereich“ (Abb. 5). Entscheidend sind eine möglichst gute Zentrierung und eine Druckverteilung, die den sensiblen Apex nur touchiert und beim Lidschlag eine ausreichende Unterspülung zulässt [36].

Gerade beim Keratokonus sollten **Routinekontrollen**

Routinekontrollen

häufig erfolgen, weil aufgrund der vielfach reduzierten Hornhautsensibilität signifikante Komplikationen verspätet wahrgenommen werden können [32].

#### Merke

Zur Vermeidung von Kontaktlinsentrageschäden empfehlen sich regelmäßige Kontrollen, am besten im Halbjahresrhythmus abwechselnd beim KL-Anpasser (mit getragener KL) und beim Augenarzt (mit 3 Tagen KL-Karenz) – bei Beschwerden jederzeit sofort.

#### Cave

Durch die heutigen breit gefächerten Möglichkeiten der Hornhautchirurgie sollte die KL-Anpassung beim KK nicht mehr „gewaltsam“ auf extreme Fälle mit häufigem Linsenverlust oder gar den kornealen Hydrops ausgeweitet werden!

### Phototherapeutische Keratektomie mittels Excimerlaser

Eine Indikation für die phototherapeutische Keratektomie mittels Excimerlaser (Excimer-PTK) beim KK sind **subepitheliale Knötchen**

subepitheliale Knötchen

oder sehr **oberflächliche Narben**

oberflächliche Narben

in der optischen Zone. Das primäre Ziel ist die Verbesserung bzw. Ermöglichung des Kontaktlinsensitzes, um so invasivere Mikrochirurgie zu vermeiden oder hinauszuzögern [37]. Die topographiegestützte PTK in Kombination mit Riboflavin-UVA-CXL [38] kann heute *nicht* als empfehlenswerte Standardbehandlung des KK erachtet werden.

#### Merke

Bei der PTK so wenig wie möglich abtragen.

#### Cave

Biomechanik.

### Riboflavin-UVA-Crosslinking

Sollte es zu einer Progredienz des KK kommen, so empfiehlt sich ein Riboflavin-UVA-Crosslinking (CXL), sofern die stromale Hornhautdicke an der dünnsten Stelle (> 400 µm) dieses zulässt und keine zentrale relevante Narbenbildung besteht [39]. Das Ziel des CXL beim Keratokonus ist es, die **Stabilität der Hornhaut**

Stabilität der Hornhaut

zu verbessern. Die Kollagenfasern im Hornhautstroma werden beim CXL durch Riboflavin und unter Einwirkung von UVA-Licht vernetzt [40]. Dies ermöglicht es, über einen längeren Zeitraum formstabile Keratokonusspezialkontaktlinsen tragen zu können oder auch den Zeitpunkt einer Keratoplastik hinauszuzögern [41]. Die Effektivität des CXL wurde in 2 prospektiven randomisierten Studien nachgewiesen [42, 43].

#### Cave

Dem Patienten darf man keine deutliche Verbesserung der Sehkraft nach CXL versprechen!

Die in Infobox 1 und 2 stehenden Kriterien des **Gemeinsamen Bundesausschusses**

Gemeinsamen Bundesausschusses

(GBA) müssen erfüllt sein, damit die Behandlung in Deutschland eine Kassenleistung ist.

#### Infobox 1 Kriterien des Gemeinsamen Bundesausschusses (GBA)

Die Hornhautvernetzung darf erbracht werden bei Patientinnen und Patienten mit Keratokonus und subjektiver Sehverschlechterung, bei denen anhand mindestens eines der folgenden Kriterien eine Progredienz innerhalb der letzten 12 Monate vor Indikationsstellung zur Hornhautvernetzung festgestellt wurde:

- Zunahme der maximalen Hornhautbrechkraft um ≥ 1 dpt,
- Zunahme des durch die subjektive Refraktion bestimmten Astigmatismus um ≥ 1 dpt,
- Abnahme der Basiskurve der bestsitzenden Kontaktlinse um ≥ 0,1 mm,

und die mittels Hornhauttomographie bestimmte Hornhautdicke an der dünnsten Stelle bei Beginn der Bestrahlung mindestens 400 μm beträgt.

#### Infobox 2 Beschluss des GBA über eine Änderung der Richtlinie Methoden vertragsärztliche Versorgung: UV-Vernetzung mit Riboflavin bei Keratokonus (§ 2 Indikationsstellung): https://www.g-ba.de/downloads/39-261-3417/2018-07-19_MVV-RL_UV-Vernetzung-Riboflavin-Keratokonus_BAnz.pdf


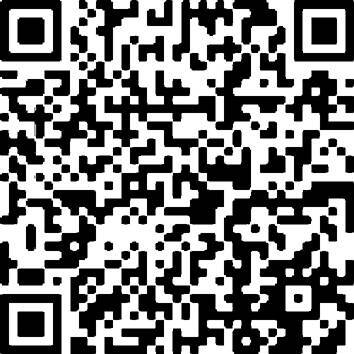


Um Aussagen zur **Progredienz**

Progredienz

**Figure 6:**
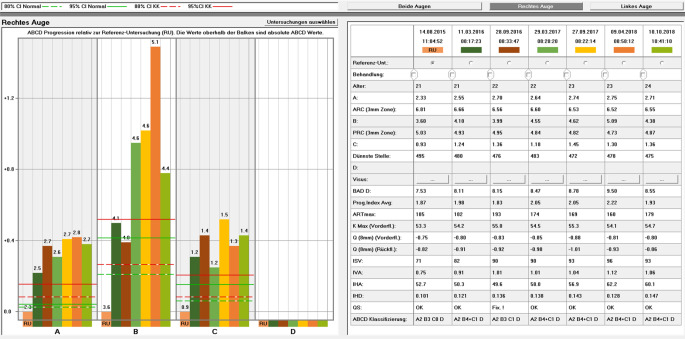

**Figure 7:**
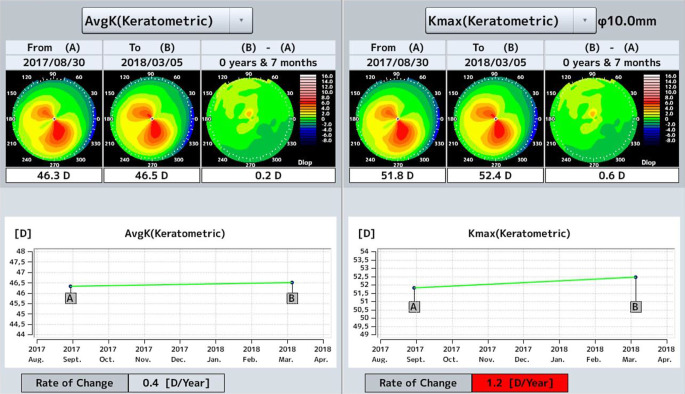

[44] des KK treffen zu können, ist es wichtig, die betroffenen Patienten im Verlauf standardisiert zu untersuchen und die Ergebnisse der jeweiligen Untersuchungen miteinander zu vergleichen. Hierzu stehen uns heute das „Belin ABCD Progressions-Display“ der Pentacam nach Michael Belin (Abb. 6; [45]) und eine „Trend Analysis“ der Vorderabschnitts-OCT CASIA-2 (Abb. 7) zur Verfügung. Um die Progredienz zwischen 2 Untersuchungen korrekt evaluieren zu können, muss man die Reproduzierbarkeit der Werte bei sequenziellen Aufnahmen kennen. Diese Reproduzierbarkeit nimmt mit zunehmendem KK-Schwergrad signifikant ab [46].

#### Merke

Besonders bei Kindern und Patienten mit Trisomie 21 ist Crosslinking eine gute Behandlungsmethode.

Nach dem „**Dresdner Protokoll**

Dresdner Protokoll

“ wird nach Oberflächenanästhesie das zentrale Epithel in einem Durchmesser von ca. 9 mm manuell entfernt („Epi-off“). Anschließend wird eine Lösung von 0,1 % Riboflavin in 20 % Dextran alle 2–3 min für 30 min eingetropft, worauf die Bestrahlung mit UVA-Licht (365–370 nm) bei 3 mW/cm^2^ für 30 min folgt (Gesamtenergie 5,4 J/cm^2^) [47]. Nach Beendigung wird eine therapeutische Kontaktlinse aufgesetzt und eine antibiotische Lokaltherapie verordnet.

Postoperativ ist nach etwa 2 Wochen besonders mittels VA(Vorderabschnitts)-OCT die sog. „**Demarkationslinie**

Demarkationslinie

“ als eine transitive Zone zwischen behandelter und unbehandelter Hornhaut darstellbar – besonders nach Epi-off-CXL [48].

Typischerweise kann die optische Kontaktlinse nach spätestens 3 Monaten wieder angepasst werden.

#### Cave

Die Festigkeitszunahme der Hornhaut nach Crosslinking *ohne* Epithelentfernung (transepitheliales oder Epi-on-CXL) erreichte in Laborstudien nur ein Fünftel bis ein Drittel der Festigkeitszunahme des CLX *mit* Epithelentfernung (Epi-off-CLX) [49].

Beim „**Accelerated Crosslinking**

Accelerated Crosslinking

“ wird Dextran in der Lösung durch Hydroxypropylmethylcellulose (HPMC) ersetzt, wodurch die Diffusionsrate verdoppelt werden kann und sich die Einwirkdauer auf 20 min reduziert. Als positive Nebeneffekte gibt es zum einen intraoperativ keine starke Verminderung der Hornhautdicke, und zum anderen wird die UVA-Belastung auf der Ebene des Endothels reduziert. Die Verkürzung der Bestrahlungsdauer mit UVA-Licht kann durch eine Erhöhung der Bestrahlungsintensität auf bis zu 9 mW/cm^2^ kompensiert werden. Die applizierte Gesamtdosis von 5,4 J/cm^2^ bleibt dabei konstant. Die Ergebnisse des „Accelerated CXL“ werden als gleichwertig oder dem Standardprotokoll nur geringfügig unterlegen eingeschätzt [50, 51]. Derzeit gibt es auf dem Markt verschiedene Geräte für das CXL mit Unterschieden in der möglichen Variation von Bestrahlungsdauer bzw. Intensität.

*Empfehlung: *Nach ausführlicher (!) Aufklärung über Chancen und Risiken (Infektion, Einschmelzung, Narben) empfehlen wir derzeit das **Epi-off-Crosslinking**

Epi-off-Crosslinking

(und nur das!) (mindestens 10 min Bestrahlungsdauer!) bei jeder nachgewiesenen Progression des KK – besonders auch bei Kindern. Intraoperativ werden sicherheitshalber 4 Messungen der zentralen Hornhautdicke empfohlen: mit Epithel, nach Abrasio corneae, vor Bestrahlung und nach Bestrahlung. Der kontaktlinsenkorrigierte Visus sollte hierbei noch zufriedenstellend sein, denn es kommt anschließend typischerweise nicht zu einer Visusverbesserung, vielmehr soll der Status quo ante „eingefroren“ werden. Erfahrungsgemäß geht das CXL jedoch auch mit einer manchmal im Verlauf zunehmenden leichten Abflachung der zentralen Hornhautkrümmung einher. Insgesamt ist das CXL aber bei schlechtem Visus und Kontaktlinsenintoleranz *keine* gute Option.

#### Cave

Das CXL macht keinen Sinn, wenn die Sehkraft schon schlecht ist!

Kontraindikationen für das CXL sind eine Hornhautdicke < 400 µm oder zentrale Hornhautnarben. Ebenso sollte bei akutem Keratokonus, Kollagenosen (z. B. Sklerodermie), schwerer Neurodermitis oder Erkrankungen aus dem rheumatischen Formenkreis von der CXL-Methode abgesehen werden [41, 47].

#### Cave

**Figure 8:**
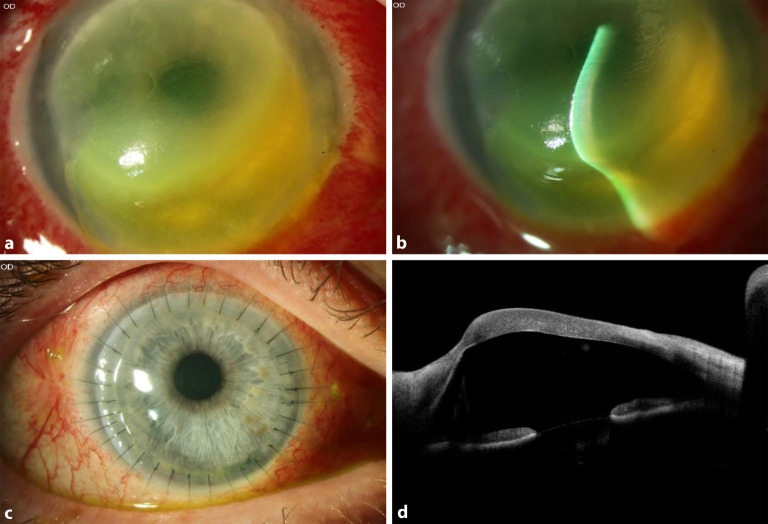

Beim CXL tritt immer eine vorübergehende Trübung („Haze“) auf. CXL kann aber auch zu einer 1) infektiösen Keratitis, 2) zentralen Vernarbung oder 3) Einschmelzung (Abb. 8a–d) führen.

Daneben sind auch eine weitere **Progression**

Progression

sowie Endothelschäden, sterile Infiltrate, persistierende Epitheldefekte möglich, oder es kann zu einer Herpesreaktivierung kommen [52]. Bei erneuter Progression kann das CXL im Intervall mindestens 1‑mal wiederholt werden [53].

#### Cave

Die zurückliegende herpetische Keratitis gilt als Kontraindikation für das CXL beim KK!

### Intrakorneale Ringsegmente

Die Implantation von intrakornealen Ringsegmenten (ICRS) wird empfohlen bei Kontaktlinsenintoleranz, unzureichendem brillenkorrigiertem Visus und ausreichend dicker mittelperipherer Hornhaut ohne zentrale Narben und einem steilen K‑Wert unter 62 dpt [54]. Hier haben sich die hexagonalen intrastromalen Hornhautimplantate mit einer optischen Zone von 6–7 mm bewährt [55]. Eine weitere Methode sind die **Ferrara-Ringe**

Ferrara-Ringe

mit einer triangulären Form und einer optischen Zone von 5–6 mm. Sie werden in Deutschland nicht bevorzugt, weil sie eher zur Induktion eines irregulären Astigmatismus neigen mit vermehrt Halos und Blendung [56].

**Figure 9:**
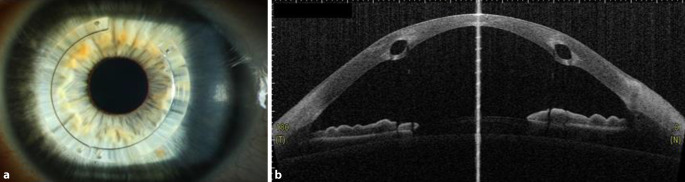

Die **intrastromalen Hornhautimplantate**

intrastromalen Hornhautimplantate

stellen eine elegante und effektive Option dar, um den unkorrigierten und bestkorrigierten Visus zu verbessern und (wie durch ein „Korsett“) auch die Progression des Keratokonus einzudämmen (Abb. 9a, b). In einer Langzeitstudie mit einem Follow-up von im Mittel 3 Jahren zeigten nur 2,7 % der Patienten unter 35 Jahre eine Progredienz des KK nach reiner INTACS-Implantation [57]. Nichtsdestoweniger ist prinzipiell eine Kombinationstherapie mit CXL möglich – besonders bei jüngeren Patienten mit stärkerer Progressionstendenz. Vor allem Patienten mit einer Intoleranz gegenüber formstabilen Kontaktlinsen profitieren von dem abflachenden – also hyperopisierenden – Effekt der intrastromalen Hornhautimplantate, sodass oftmals bei „regularisiertem Astigmatismus“ wieder eine Brillen- oder Kontaktlinsenanpassung möglich wird.

#### Merke

Intrakorneale Ringsegmente können den sc-Visus und den cc-Visus beim Keratokonus verbessern.

Voraussetzung für die Implantation von ICRS sind eine Hornhautdicke von > 450 µm in der 6‑ bis 7‑mm-Implantationszone der Vorderabschnitts-OCT und eine narbenfreie optische Zone. Die zentrale Hornhautdicke spielt hierbei eher eine untergeordnete Rolle. Die Lage, Dicke und Geometrie der Halbringe wird individuell entsprechend dem Nomogramm der Herstellerfirma angepasst.

Die Ringsegmente werden in 2 Schritten implantiert. Zuerst erfolgt eine Tunnelpräparation mittels Femtosekunden(FS)-Laser in 80 % mittelperipherer Hornhauttiefe, und anschließend werden die Ringsegmente mittels chirurgischer Spezialpinzette implantiert. Da keine mechanische Tunnelkreation mehr erfolgt, werden eine Dezentrierung des Segments, inadäquate Tunneltiefe und -breite, oberflächliche Tunneldissektion mit Perforation der Bowman-Lamelle nach außen sowie eine Perforation in die Vorderkammer bei der Tunnelkreation oder beim Vorschieben des Ringsegments weitgehend vermieden [56]. Zu den klaren Vorteilen der **FS-lasergestützten ICRS-Implantation**

FS-lasergestützten ICRS-Implantation

zählen die geringe Invasivität der Behandlung mit einem sehr überschaubaren Komplikationsrisiko sowie die Reversibilität der Prozedur.

#### Merke

Die Tunnelkreation für die ICRS sollte heute ausschließlich mit dem Femtosekundenlaser erfolgen.

### Akuter Keratokonus (= kornealer Hydrops)

Beim akuten Keratokonus (sog. **Hydrops corneae**

Hydrops corneae)

tritt aufgrund von Überdehnung ein strichförmiger Defekt („Riss“) in der Descemet-Membran und Dua-Schicht auf. Die Prävalenz des kornealen Hydrops beim Keratokonus liegt bei 1,6–2,8 % [58]. Ein höheres Risiko soll für Ostasiaten und dunkelhäutige KK-Patienten bestehen. Als Prädisposition gelten: Augenreiben, Atopie, Allergie, Keratoconjunctivitis vernalis und Trisomie 21. Der Trigger ist oft ein Hornhauttrauma, exzessives Reiben mit den Fingergelenken oder schwerer Husten [59].

Ein Hydrops corneae ist üblicherweise innerhalb von 3 bis 6 Monaten selbstlimitierend. Anschließend ist aufgrund einer narbenbedingten Visusminderung häufig eine perforierende Keratoplastik (PKP) notwendig. Allerdings besteht auch die Möglichkeit des Visusanstiegs durch eine vernarbungsbedingte Abflachung und topographische Regularisierung, wenn die Narbe (mittel)peripher liegt.

#### Cave

Im Akutstadium des Keratokonus ist eine Keratoplastik à chaud kontraindiziert!

Über viele Jahrzehnte bestand in Deutschland die allgemein anerkannte „Therapie“ in Abwarten unter Gabe von 5 % hypertonen Natriumchloridtropfen, Pflege und ggf. Drucksenkung. Im Gegensatz zu früher legen wir heute stattdessen routinemäßig multiple tiefstromale – im Idealfall prädescemetale – **10-0-Nylon-Nähte**

**Figure 10:**
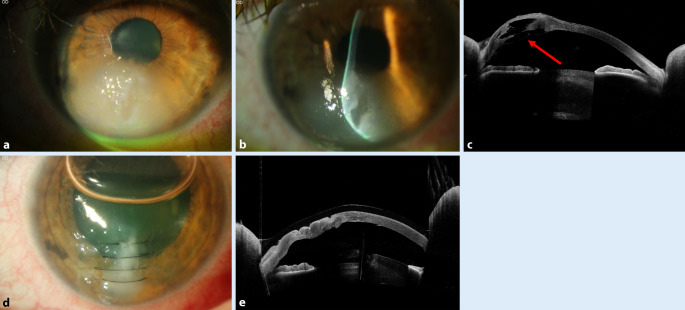

10-0-Nylon-Nähte

senkrecht zum Riss in Tropfanästhesie, um so zusammen mit einer Luftblase (bevorzugt nach kaudaler YAG-Iridotomie) eine sehr schnelle Reduktion des Stromaödems zu erreichen [60, 61, 62]. Die Luft in der Vorderkammer soll zusätzlich den Wassereinstrom in das korneale Stroma vermeiden (Abb. 10a–e, Infobox 3). Zur präoperativen Lokalisation des Descemet-Risses kann die Vorderabschnitts-OCT trotz starker ödematöser Trübung hilfreich sein [63].

#### Infobox 3 Operatives Vorgehen zum Legen von Muraine-Nähten: https://vimeo.com/444156902


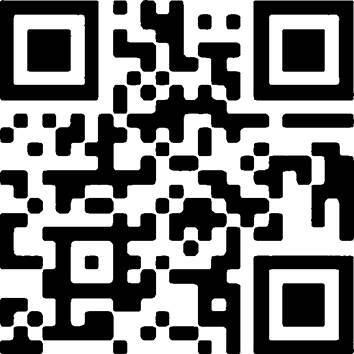


Bei der Kontrolluntersuchung 6 Wochen postoperativ zeigt sich typischerweise eine abgeheilte reizfreie Hornhaut mit einer regulären Dicke. Zu diesem Zeitpunkt entfernen wir alle Fäden in Tropfanästhesie. Jeder lockere Hornhautfaden wird – wie immer – vorher sofort entfernt!

#### Merke

Multiple tief-stromale 10-0-Nylon-Einzelknüpfnähte senkrecht zum Descemet-Riss führen rasch zu einer Entquellung des Stromas beim kornealen Hydrops.

**Figure 11:**
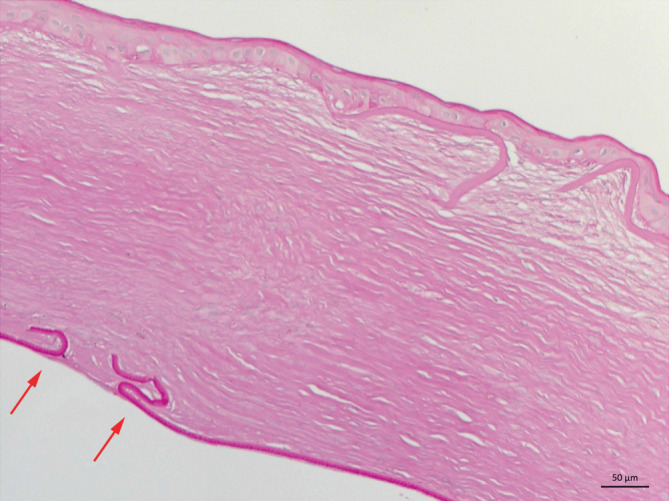

**Figure 12:**
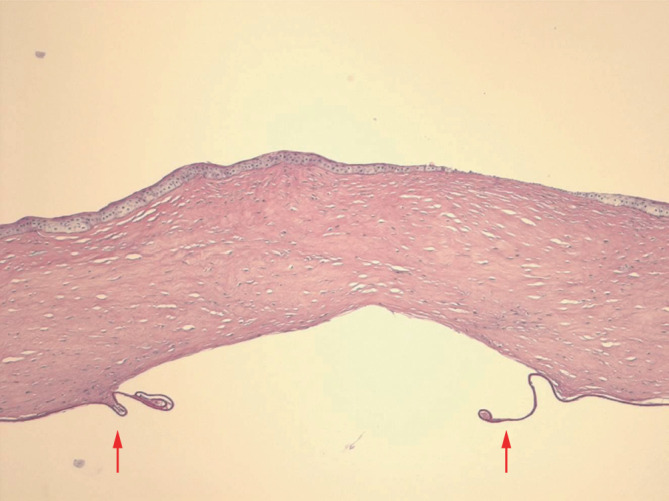

Die **histopathologische Aufarbeitung**

histopathologische Aufarbeitung

unserer Hornhautexzisate nach akutem Keratokonus mit vs. ohne sog. „Muraine-Nähte“ zeigt beispielhaft einen signifikant geringeren Abstand der Descemet-Ränder von etwa 70 µm (Abb. 11) nach sog. „Muraine-Nähten“ im Vergleich zu etwa 1400 µm (Abb. 12) nach klassischer Spontanheilung [59].

Alternativ wurde jüngst von der Kölner Arbeitsgruppe eine **Mini-DMEK**

Mini-DMEK

(Descemet-Membran endotheliale Keratoplastik) vorgeschlagen, um bei sehr dünnen Hornhäuten den Descemet-Defekt abzudecken [64]. So könne der Austritt von Kammerwasser durch die mitunter auch durchgreifenden Stichkanäle der „Muraine-Nähte“ mit abgeflachter Vorderkammer vermieden werden.

Eine PKP kann so entweder vermieden werden oder bei KL-Intoleranz schneller erfolgen, ohne dass mit Fadenlockerungen wie nach Keratoplastik im Akutstadium des Hydrops zu rechnen ist. Das hat v. a. bei jungen KK-Patienten große Bedeutung für die Berufs- und gesamte Lebensplanung.

### Perforierende und lamelläre Hornhauttransplantation

Sollte die Hornhaut bereits zu dünn sein oder sollten bereits visusrelevante Narben vorliegen und damit eine Kontraindikation für ICRS bestehen, so ist die Hornhauttransplantation in diesem Stadium der Erkrankung indiziert und weist eine sehr gute Prognose von bis zu 95 % auf.

### Perforierende Keratoplastik

Bei fortgeschrittenem KK mit tief-stromaler Vernarbung (besonders nach akutem KK mit Descemet-Defekt) bleibt die PKP die Methode der Wahl. Typischerweise lassen sich mit dieser Methode die (para)zentralen verdünnten, irregulären und vernarbten Hornhautanteile komplett entfernen [65]. Eine **optimale Transplantatgröße**

optimale Transplantatgröße

sollte individuell angepasst werden, wobei hier gilt: „So groß wie möglich, so klein wie nötig“ [66]. Bei der Operation ist die **optimale Lagerung**

optimale Lagerung

(horizontale Kopfposition und horizontale Limbusposition) sehr wichtig. Eine optimale Zentrierung der Exzision orientiert sich typischerweise am Limbus aufgrund der zu erwartenden optischen Verlagerung der sichtbaren („entrance“) Pupille beim Keratokonus [67]. Zur Vermeidung eines Urrets-Zavalia-Syndroms mit persistierender weiter Pupille aufgrund einer druckbedingten Iris-Sphinkter-Nekrose in Atropin-Mydriasis sollte intraoperativ routinemäßig eine **Open-sky-Iridotomie**

Open-sky-Iridotomie

bei 12 Uhr erfolgen [68].

### Konzept der Excimerlaser-assistierten perforierenden Keratoplastik

Seit mehr als 30 Jahren steht die nichtmechanische Excimerlaser-assistierte PKP mit 8 maskenvermittelten **Orientierungszähnchen**

Orientierungszähnchen

/-﻿kerben zur Verfügung, wodurch die Kompression und Distorsion von Hornhautgewebe während der Trepanation vermieden werden [69]. Die Anwendung der Orientierungszähnchen hat in erster Linie den praktischen Vorteil für den Mikrochirurgen, dass die exakte Position der zweiten Situationsnaht eindeutig vorgegeben ist.

Bei Transplantatdurchmessern von 8,0 bzw. 8,5 mm wird damit im Mittel ein Brillen(!)-Visus von 0,8 nach Fadenentfernung erreicht [70]. Ein neuartiger **Kreuzstichmarker**

**Figure 13:**
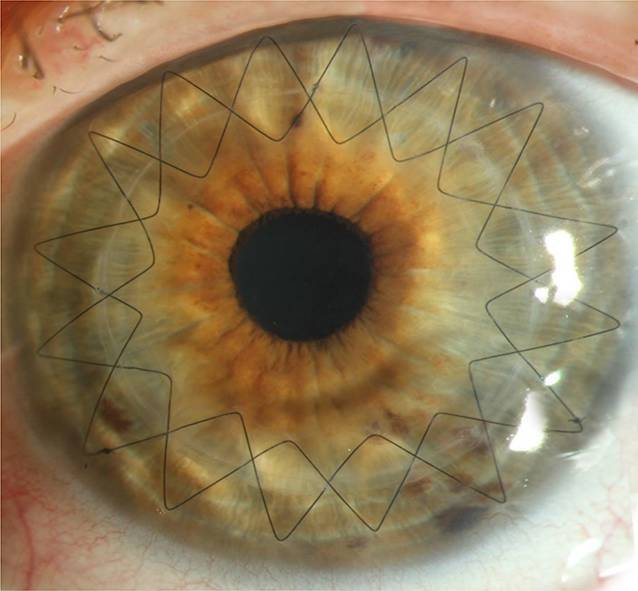

**Figure 14:**
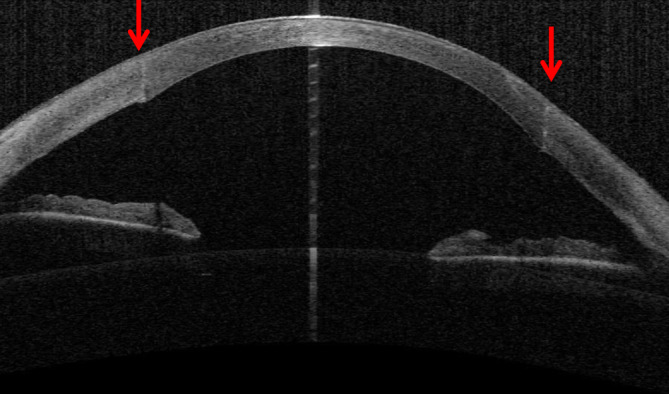

**Figure 15:**
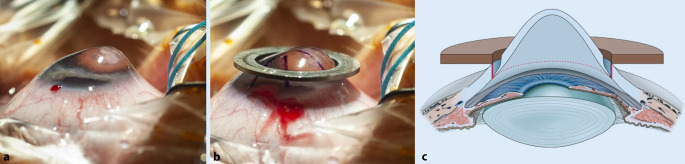

Kreuzstichmarker

erlaubt es auch dem weniger erfahrenen Hornhautchirurgen, eine regelmäßige doppelt fortlaufende Kreuzstichnaht nach Hoffmann zu legen ([71]; Abb. 13). Der Astigmatismus und die Regularität der Topographiewerte sind nach Fadenentfernung wegen optimaler Spender-Empfänger Apposition (Abb. 14) signifikant günstiger als nach mechanischer Trepanation [72]. Die funktionellen Ergebnisse nach **kontaktfreier Excimerlaser-PKP**

kontaktfreier Excimerlaser-PKP

sind bei Operation im fortgeschrittenen Stadium oder bei vernarbtem akutem Keratokonus nicht schlechter als bei Operation in früheren Stadien [73]. Besonders bei notwendiger Rekeratoplastik wegen kleinen (dezentrierten) primären Transplantats mit hohem irregulärem Astigmatismus (sog. „Keratokonusrezidiv“) erlaubt die zentrierte 8,5/8,6-mm-Excimerlaser-assistierte Retrepanation oft, das gesamte alte Transplantat inklusive der Spender-Empfänger-Appositionsnarbe kontaktfrei zu exzidieren [74]. Diese Variante lässt in Kombination mit Einzelknüpfnähten bei geringer Dezentrierung nach unten auch die PKP bei fortgeschrittener PMD (= Keratotorus) mit kaudal sehr dünner (mittel)peripherer Hornhaut zu (Abb. 15a–c).

#### Merke

Die Excimerlaser-PKP führt beim Keratokonus zu geringem Astigmatismus, höherer Regularität in den Topographieergebnissen und besserem Brillenvisus.

### Nachteile der Femtosekundenlaser-assistierten perforierenden Keratoplastik

Bei der FS-assistierten PKP wegen KK kommt es zu mehr Dezentrierung, mehr „Vis à tergo“, und es sind öfter Einzelknüpfnähte nötig, um eine Spender-Empfänger-Apposition ohne Stufen und Lücken zu erreichen [75]. Außerdem ist der **objektive Astigmatismus**

objektive Astigmatismus

nach Fadenentfernung signifikant höher als nach Excimerlasertrepanation [76] und als nach freihändiger Trepanation [77]. Das liegt unter anderem daran, dass durch die Notwendigkeit der Ansaugung und Applanation der Kornea keine runde, sondern eine elliptische oder birnenförmige Öffnung in die instabile KK-Patientenhornhaut geschnitten wird. Das führt zu einer teils gravierenden Inkongruenz von Transplantats- und Wirtsgeometrie und einem hohen und/oder irregulären Astigmatismus – spätestens nach Fadenentfernung. Die FS-assistierte Trepanation muss somit wegen diverser gravierender Nachteile (Tab. 2) als das „excitement of yesterday“ in der kurativen Mikrochirurgie des KK gelten [78].

**Table 2:** 

Lasertyp	Excimer	Femtosekunden
„Umständliche Prozedur“	+	− −
Zentrierung	+ + +	+
Vermeidung von Deformierung und Kompression	+ + +	− − −
Hoher IOD während der Laseraktion	+ + +	−
Minimierung der Schnittkomplettierung mit Schere	(+)	+ +
Eindeutige Lokalisation der ersten 8 Situationsnähte	+ + +	+
Stabile Vorderkammer während der Naht	+ +	+ + +
Möglichkeit einer doppelt fortlaufenden Naht	+ + +	+ + +
Keine Notwendigkeit zusätzlicher EKN	+ + +	+
Möglichkeit der Trepanation bei instabiler Kornea	+ + +	− − −

+ + + sehr gut, − − − sehr schlecht, *IOD* intraokularer Druck, *EKN* Einzelknüpfnaht

#### Cave

Die Femtosekundenlaserkeratoplastik führt aufgrund der Notwendigkeit der Applanation während der Trepanation zu sehr hohen Astigmatismuswerten nach Fadenentfernung und sollte deshalb in der Kontaktvariante vermieden werden.

### „Deep anterior lamellar keratoplasty“

Alternativ kann die vordere lamelläre Keratoplastik („deep anterior lamellar keratoplasty“ [DALK]) bei prädescemetaler Narbenfreiheit und gutem Endothel in geübten Händen eine probate Option darstellen [79, 80]. Potenzielle **DALK-Indikationen**

DALK-Indikationen

sind besonders bei jungen Patienten mit Neurodermitis neben dem KK auch die PMD oder stromale Hornhautdystrophien ohne Endothelbeteiligung [79]. Es gilt zu beachten, dass mit zunehmendem Schweregrad des KK die Endothelzellzahl signifikant sinkt und der Pleomorphismus/Polymegalismus der Endothelzellen signifikant zunimmt [81]. Diese Erkenntnisse sollten bei der Indikationsstellung zur DALK bei fortgeschrittenem KK bedacht werden. Insgesamt wird die DALK in Deutschland nach einer Erhebung der DOG(Deutsche Ophthalmologische Gesellschaft)-Sektion Kornea nur in 2–3 % aller Keratoplastiken angewendet [82].

#### Merke

Das Hornhautendothel muss für eine DALK intakt sein.

Die DALK hat ein **geringeres Abstoßungsrisiko**

geringeres Abstoßungsrisiko,

da sich die häufigste und gravierendste Immunreaktion gegen das Endothel der Spenderhornhaut richtet. Anwar und Teichman schlugen erstmals 2002 die **Big-Bubble-Technik**

Big-Bubble-Technik

für die DALK vor, um die pure Descemet-Membran vom Stroma zu trennen [83]. Grundsätzlich sollte die intendierte DALK nur als solche zu Ende geführt werden, wenn intraoperativ – bevorzugt mittels Big-Bubble-Technik – die Descemet-Membran freigelegt und nicht perforiert wird, um ein gutes Visusergebnis erzielen zu können (Tab. 3).

**Table 3:** 

Parameter	Operation
Expulsive Blutung	DALK ≪ PKP
Endothelzellverlust	DALK < PKP
Immunreaktionen	DALK ≪ PKP
Wundstabilität	DALK > PKP
Astigmatismus	DALK = PKP
Fadenlockerung	DALK > PKP
Stromale Vaskularisation	DALK > PKP
Operationsdauer	DALK ≫ PKP
Akuter Keratokonus	DALK ⋘ PKP
*Visus*	*DALK* < *PKP*

#### Cave

Keine DALK bei Zustand nach akutem Keratokonus mit prädescemetalen Narben!

In allen anderen Fällen sollte bei diesen jungen Patienten lieber zur PKP konvertiert werden. Die **Konversionsrate**

Konversionsrate

lag in einer neueren Studie aus Italien bei 16,2 % [84]. In einer Studie mit multiplen Operateuren aus England lag die intraoperative Perforationsrate der Descemet-Membran bei manueller Technik bei 45,4 % (Konversionsrate zu PKP 24,5 %) im Gegensatz zu 25,9 % bei FS-gestützter DALK (Konversionsrate zu PKP 3,4 %) [85]. Diese Details müssen bei der Aufklärung zur DALK stets Berücksichtigung finden!

#### Cave

Der mittlere Visus im Langzeitverlauf nach DALK liegt typischerweise bei „20/40“ (d. h. die Hälfte der Patienten sieht postoperativ weniger als 0,5!).

### Konzept der Excimerlaser-assistierten „deep anterior lamellar keratoplasty“

Beim **Homburger Konzept**

Homburger Konzept

der Excimerlaser-assistierten DALK wird die reguläre Spendergewinnung mittels Excimerlasertrepanation mit „Zähnchen“ vorgenommen [86]. Beim Patienten erfolgt eine tiefe lamelläre Excimerlasertrepanation mit „Kerben“ (80 % der mit der VA-OCT gemessenen mittelperipheren Hornhautdicke [46]). Dann wird eine dicke vordere Stromalamelle manuell entfernt. Gelingt dann die „Big Bubble“ in den dünnen posterioren Stromaschichten, wird die Operation als DALK beendet [86]. Andernfalls kann der Operateur bei der Excimerlaser-assistierten DALK zur Excimerlaser-assistierten PKP „konvertieren“ – mit vertretbarem zeitlichem Aufwand und ohne Nachteil für die oft jungen Patienten, sofern primär ein Spendergewebe mit gutem Endothel vorgesehen wurde.

#### Merke

Die Excimerlaser-assistierte DALK kombiniert die technischen Vorteile für den Mikrochirurgen und reduziert die Nachteile für den Patienten im Falle der Notwendigkeit der Konversion.

Es wird am Ende der Operation routinemäßig eine **Luftblase**

Luftblase

(etwa 80 %) in die Vorderkammer gegeben, um eine Anhaftung der Descemet-Membran des Patienten an das Spenderstroma sicherzustellen und um eine sog. „doppelte Vorderkammer“ zu vermeiden. Um einer Augeninnendruckerhöhung vorzubeugen („Luftblock“), sollte routinemäßig präoperativ – analog zur DMEK [87] – eine **Nd:YAG-Laser-Iridotomie**

Nd:YAG-Laser-Iridotomie

bei 6 Uhr angelegt werden.

#### Cave

Immer Nd:YAG-Laser-Iridotomie bei 6 Uhr vor DALK zur Vermeidung eines Luftblocks mit Augeninnendruckentgleisung!

### Zentrale Korneoskeralplastik

**Figure 16:**
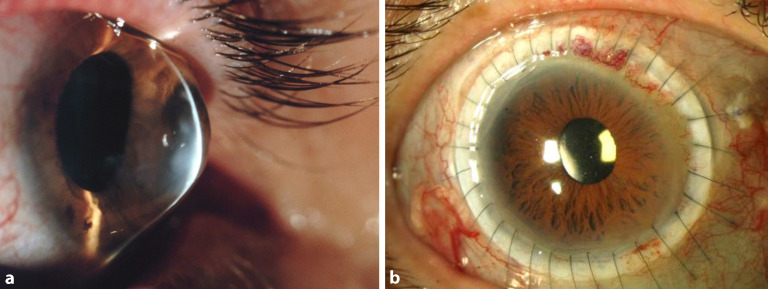

Bei einem bilateralen Keratoglobus oder einer PMD (= Keratotorus) mit deutlicher Progredienz und KL-Intoleranz (auch skleragestützt in der Hand eines Spezialisten!) kann die zentrale 12-mm-Korneoskleralplastik eine Ultima-Ratio-Option sein (Abb. 16a, b). Eine **systemische Immunsuppression**

systemische Immunsuppression

(Ciclosporin A oder Mycophenolat-Mofetil) für mindestens 1 Jahr unter monatlicher internistischer Blutbildkontrolle ist dabei obligat. Das Transplantat besteht aus einem Hornhautanteil (volle Dicke) und einem skleralen Anteil (½ Dicke) [65].

#### Cave

Bei der zentralen Korneoskleralplastik ist die intraoperative Schonung des Skleralsporns zur Vermeidung eines partiellen Winkelblockglaukoms obligat!

## Fazit für die Praxis

- Der Keratokonus (KK) kann heute bereits im „Früheststadium“ diagnostiziert werden und so gut wie kaum eine andere Hornhauterkrankung Erfolg versprechend einer guten stadiengerechten Therapie zugeführt werden.
- Das ABCD-Grading-System nach Belin dient beim KK als leicht zu verinnerlichende Klassifikation zur Bestimmung des Schweregrades und zur Dokumentation der Progression im Verlauf.
- Nach wie vor sind O_2_-durchlässige, formstabile Kontaktlinsen die erste Wahl zur refraktiven Korrektur des KK.
- Das Crosslinking (CXL) hat heute einen wichtigen Stellenwert bei der Stabilisierung des KK bei nachgewiesener Progression und noch gutem Visus.
- Die Femtosekundenlaser-assistierte ICRS(intrastromale Ringsegmente)-Implantation hat heute eine Nische bei mäßiggradigem Konus, klarer zentraler Hornhaut und Kontaktlinsenintoleranz erobert.
- Bei akutem KK sollte keine Keratoplastik durchgeführt werden, sondern die Verwendung von tief-stromalen Kompressionsnähten mit Lufttamponade (nach dem Erstbeschreiber sog. „Muraine-Nähte“) in Betracht gezogen werden.
- Die DALK („deep anterior lamellar keratoplasty“, bevorzugt Excimerlaser-assistiert) kann eine Option sein, wenn die Descemet-Membran freigelegt wird und damit Visusergebnisse von 0,8 bis 1,0 erreichbar sind.
- Dagegen ist die zentrale runde 8,0- bzw. 8,5-mm-PKP (perforierende Keratoplastik, bevorzugt Excimerlaser-assistiert) mit doppelt fortlaufender Kreuzstichnaht nach Hoffmann heute immer noch State-of-the-Art beim fortgeschrittenen KK (besonders nach akutem KK).
- Bei bilateralem Keratoglobus mit maximaler peripherer kornealer Verdünnung und Skleralkontaktlinsenintoleranz kann die zentrale ~ 12-mm-Korneoskleralplastik eine Ultima Ratio zur partiellen visuellen Restitution darstellen.

## CME-Fragebogen

Ein junger Patient mit Keratokonus stellt sich zur Kontrolle mit einem stabilen cc(cum correctione)-Brillenvisus von 0,1 am RA (rechtes Auge) und 0,4 am LA (linkes Auge) vor. Der Visus lässt sich beidseits mit formstabilen Kontaktlinsen auf 0,7 bessern, der Patient verträgt aber im Rahmen seiner Arbeit als Bauarbeiter keine Kontaktlinse. Die Hornhäute zeigen keine Narbe, die dünnste Hornhautdicke beträgt rechts 450 µm und links 430 µm, die maximale Vorderflächenkrümmung (K_max_) beträgt beidseits unter 65 dpt. Welche therapeutische Option ist für das rechte Auge primär zu bevorzugen?

Forcierter Versuch mit Kontaktlinse („Stellen Sie sich nicht so an!“)

„Accelerated Crosslinking“

ICRS(intrastromale Ringsegmente)-Implantation

Tiefe anteriore lamelläre Keratoplastik (DALK)

Perforierende Excimerlaser-assistierte Keratoplastik (PKP)

Bei welchem Verfahren zur Therapie des Keratokonus empfiehlt sich eine intraoperative Iridotomie bei 12 Uhr, um ein Urrets-Zavalia-Syndrom zu vermeiden?

Riboflavin-UVA-Crosslinking (CXL)

Tiefstromale sog. „Muraine-Nähte“ bei akutem Keratokonus

Tiefe anteriore lamelläre Keratoplastik (DALK)

Perforierende Excimerlaser-assistierte Keratoplastik (PKP)

Descemet-Membran endotheliale Keratoplastik (DMEK) bei akutem Keratokonus

In welcher Tiefe sollten intrakorneale Ringsegmente (ICRS) im Idealfall implantiert werden?

300 µm

40 % der mittelperipheren Hornhautdicke

60 % der zentralen Hornhautdicke

80 % der mittelperipheren Hornhautdicke

Die Implantationstiefe ist für das Ergebnis nicht wesentlich.

Ab welcher Hornhautdicke muss bei Durchführung eines Riboflavin-UVA-Crosslinkings nicht mehr mit einem Endothelschaden gerechnet werden?

> 100 µm

> 200 µm

> 300 µm

> 400 µm

Die Hornhautdicke ist bei der Vernetzung kein Risikofaktor für das Auftreten eines Endothelschadens.

Welche der Folgenden ist eine allgemein akzeptierte Indikation für die Durchführung eines CXL (Crosslinking) beim Keratokonus?

Manifeste Progression des Keratokonus mit einer zentralen Hornhautdicke von 300 µm

Stabile K(Keratometrie)‑Werte über 4 Jahre bei einem 30-jährigen Patienten mit Trisomie 21

Zunahme der Hornhautbrechkraft (besonders K_max_)/des Astigmatismus um 1 dpt oder Abnahme der Basiskurve der Kontaktlinse um 0,1 mm innerhalb eines Jahres

Abfall des bestkorrigierten Visus bei einem 18-jährigen Patienten

Zunahme der Vogt-Linien im Zentrum der Kornea

Wie hoch ist die 1‑Jahres-Transplantatüberlebensrate nach perforierender Keratoplastik bei Keratokonus?

50–60 %

60–70 %

70–80 %

80–90 %

> 90 %

Wobei wird die Implantation von intrakornealen Ringsegmenten (ICRS) empfohlen?

Guter Toleranz formstabiler KL (Kontaktlinsen)

Ausreichendem brillenkorrigiertem Visus

Deutlicher Verdünnung der mittelperipheren Hornhaut

Zentralen Hornhautnarben

Herabgesetztem Visus, klarer zentraler Hornhaut und Kontaktlinsenintoleranz

Was ist Voraussetzung für die Implantation von intrakornealen Ringsegmenten (ICRS)?

Eine Hornhautdicke von > 450 µm in der 6‑ bis 7‑mm-Zone der Vorderabschnitts-OCT (optische Kohärenztomographie)

Eine narbenreiche optische Zone

Die zentrale Hornhautdicke muss > 300 µm sein.

Pelluzide marginale Degeneration (PMD) oder Keratoglobus als Diagnose

Voraussetzung ist die Progression (Zunahme des Astigmatismus um 1 dpt oder Abnahme der Basiskurve der Kontaktlinse um 0,1 mm innerhalb eines Jahres).

Was kann nach der Keratoplastik typischerweise mit Fluorescein und Blaulicht an der Spaltlampe erkannt werden?

Tiefe der Hornhautfäden

Endothelzellpolymorphismus

Höhe des Astigmatismus

Leckagen aus der Vorderkammer

Fokale Defekte der Bowman-Lamelle

Welches ist die ungünstigste Indikation für eine Excimerlaser-gestützte DALK („deep anterior lamellar keratoplasty“)?

Pelluzide marginale Degeneration (PMD)

Zustand nach akutem Keratokonus

Post-LASIK(Laser-in-situ-Keratomileusis)-Keratektasie

Instabile Hornhaut nach radialen Keratotomien

Granuläre Hornhautdystrophie Typ 2
